# Interpreting Supervised Machine Learning Inferences in Population Genomics Using Haplotype Matrix Permutations

**DOI:** 10.1093/molbev/msaf250

**Published:** 2025-10-06

**Authors:** Linh N Tran, David Castellano, Ryan N Gutenkunst

**Affiliations:** Genetics Graduate Interdisciplinary Program, University of Arizona, Tucson, AZ 85721, USA; Department of Molecular & Cellular Biology, University of Arizona, Tucson, AZ 85721, USA; Department of Molecular & Cellular Biology, University of Arizona, Tucson, AZ 85721, USA; Department of Molecular & Cellular Biology, University of Arizona, Tucson, AZ 85721, USA

## Abstract

Supervised machine learning methods, such as convolutional neural networks (CNNs), that use haplotype matrices as input data have become powerful tools for population genomics inference. However, these methods often lack interpretability, making it difficult to understand which population genetics features drive their predictions—a critical limitation for method development and biological interpretation. Here, we introduce a systematic permutation approach that progressively disrupts population genetics features within input test haplotype matrices, including linkage disequilibrium, haplotype structure, and allele frequencies. By measuring performance degradation after each permutation, the importance of each feature can be assessed. We applied our approach to three published CNNs for positive selection and demographic history inference. We found that the positive selection inference CNN ImaGene critically depends on haplotype structure and linkage disequilibrium patterns, while the demographic inference CNN relies primarily on allele frequency information. Surprisingly, another positive selection inference CNN, disc-pg-gan, achieved high accuracy using only simple allele count information, suggesting its training regime may not adequately challenge the model to learn complex population genetic signatures. Our approach provides a straightforward, model-agnostic, and biologically-motivated framework for interpreting any haplotype matrix-based method, offering insights that can guide both method development and application in population genomics.

## Introduction

In recent years, machine learning methods have seen tremendous growth in population genetics (see Schrider and Kern 2018; Korfmann et al. 2023 and Huang et al. 2024 for recent reviews). Classic population genetics inference approaches are based on theory-informed summary statistics, but the rapid advancement of computational capabilities and sophisticated simulators has primed the field for a new paradigm: using supervised machine learning algorithms that automatically extract informative data features. The most popular among these algorithms are convolutional neural networks (CNNs), which have been applied to a wide variety of population genetics inference tasks: inferring recombination rates and hot spots (Chan et al. 2018; Flagel et al. 2019), demographic history (Flagel et al. 2019; Sanchez et al. 2021), hybrid speciation and admixture (Blischak et al. 2021), geographic dispersal (Smith et al. 2023), detecting signature of introgression (Flagel et al. 2019; Gower et al. 2021) and natural selection (Flagel et al. 2019; Torada et al. 2019; Qin et al. 2022).

Classic population genetics approaches use summary statistics that capture informative features of genomic data to make inferences. For example, various summary statistics that capture patterns of linkage disequilibrium (*D*, $D^{\prime }$, $r^{2}$) (Lewontin 1964; Hill and Robertson 1968) and haplotype structure (EHH, iHS) (Sabeti et al. 2002; Voight et al. 2006) have been devised to detect signatures of positive selection. For demographic history inference, changes in the abundance of alleles segregating at different frequencies in the population can be consolidated into the allele frequency spectrum (AFS) to offer informative signals (Fisher 1931). The AFS can be used directly as a summary statistic itself (Marth et al. 2004), or it can be further summarized by statistics such as Tajima’s D (Tajima 1989) and Fay and Wu’s H (Fay and Wu 2000). More recently, deep learning methods have also been designed to combine summary statistics as inputs into neural networks (Sheehan and Song 2016; Sanchez et al. 2021; Quelin et al. 2025).

In contrast to approaches based on summary statistics, the new CNN-based approaches aim to use the raw genomic data directly via a haplotype matrix. In this 2D matrix, haplotypes are represented as rows and segregating sites as columns. Using haplotype matrices to train machine learning models has been speculated to potentially identify new genomic features that aren’t captured by existing summary statistics (Cecil and Sugden 2023). In this spirit, Sanchez et al. (2021) designed a method that uses CNN outputs as a set of summary statistics for inference.

However, few studies have explicitly tested whether models trained on haplotype matrices have learned known features captured by traditional summary statistics, let alone new features. A closely related question, often of critical interest to genetics researchers (Musolf et al. 2022) is: what features in the data are responsible for a trained model’s prediction? In machine learning, a common method to evaluate how much a feature contributes to a model’s predictive performance is permutation-based feature importance (PFI). First introduced by Breiman (2001a, 2001b), it has since been refined and generalized by newer methods such as conditional permutation importance (CPI) (Strobl et al. 2008; Debeer and Strobl 2020), permutation importance (PIMP) (Altmann et al. 2010), and model class reliance (MCR) (Fisher et al. 2019). The general framework of PFI involves permuting the values of a given feature among the test datasets and observing the resulting degradation in model performance. The permutation breaks the relationship between the feature and the output. Thus, the more important the feature, the more model performance degrades when that feature is permuted.

In population genetics, applying permutation-based feature importance is straightforward for machine learning models based on summary statistics (Quelin et al. 2025), but not for models based on haplotype matrices. For models like CNNs, which perform automatic feature extraction from haplotype matrices, it is unclear which part of an input haplotype matrix constitutes a feature to be permuted. The shuffling of whole haplotype matrices, individual haplotypes (rows), or individual segregating sites (columns) among test datasets is not meaningful or interpretable.

Here, we introduce a method to permute test haplotype matrices in ways that are meaningful and interpretable. Instead of permuting features among test instances as in PFI, we permute entries *within* haplotype matrices in ways that sequentially disrupt interpretable features in population genetics such as linkage disequilibrium (LD), haplotype structure, and allele frequencies. Our method extends the intuitive and model-agnostic strengths of PFI to machine learning models based on haplotype matrices in population genetics. We demonstrate the power of our approach by applying it to three published CNNs trained for various population genetics inference tasks: Riley et al. (2024) (disc-pg-gan) and Torada et al. (2019) (ImaGene) for detecting signatures of positive selection, and Flagel et al. (2019) for inferring population demographic history. Our method reveals which population genetics features are important for these CNNs to achieve their inference accuracy, guiding model interpretation and future development.

## Methods

### The Haplotype Matrix Permutation Feature Importance Method

**Fig. 1.**
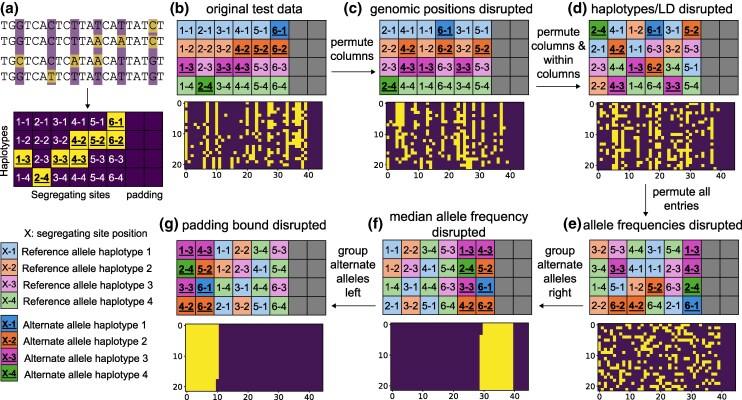
**The haplotype matrix representation of genetic variation data and the permutations to evaluate feature importance.** a) Four haplotypes are aligned, with segregating reference alleles indicated by purple and alternate alleles by yellow. In the resulting haplotype matrix, zeros are represented by purple and ones by yellow, including zero-padding. For illustration, each entry is labeled by its genomic position (column number) and haplotype (row number). b) The original illustrative test haplotype matrix is further labeled, with colors indicating haplotypes, shading and bolding representing alternate alleles, and gray representing padding. Also shown is a larger haplotype matrix that will undergo similar permutations. c) Permuting columns of segregating sites disrupts genomic positions. d) Permuting columns then entries within each column disrupts haplotypes and linkage disequilibrium. e) Permuting all entries disrupts the allele frequency spectrum (AFS). f) Grouping entries, while maintaining zero-padding, disrupts the median allele frequency. g) Grouping entries, while obscuring zero-padding, disrupts the implied number of segregating sites.

A common representation of population genetic data is a 2D haplotype matrix, in which rows are haplotypes and segregating sites are columns, ordered according to their positions in the genome (Fig. 1a&b). Typically, the matrix is binary, with 0 representing reference/major/ancestral alleles and 1 representing alternate/minor/derived alleles, depending on the specific application. Because CNNs typically require a fixed-size input data matrix, haplotype matrices are often padded with 0s in preprocessing, if the number of segregating sites is less than the input matrix size (Fig. 1a&b). Some models also use the genomic or recombination distances between segregating sites, which are input as a separate vector and which we do not permute.

In our approach, increasingly disruptive permutations are applied to haplotype matrices in the test data, and model performance is evaluated on the permuted matrices. (The trained model is not changed.) Each permutation corresponds to disrupting a set of population genetics features. Thus, if model performance degrades sharply after a specific permutation, those population genetics features are deemed important to model performance.

Our first haplotype matrix permutation is to randomly permute whole non-padding columns (each corresponding to a segregating site) among positions in the matrix (Fig. 1c). This disrupts the original genomic positions of the segregating sites. Second, we randomly permute whole non-padding columns and also randomly permute entries within each column (Fig. 1d). This destroys correlations between neighboring segregating sites, disrupting haplotypes and LD features. Third, we randomly permute all non-padding entries in the haplotype matrix (Fig. 1e). This disrupts the AFS, because observed allele frequencies become binomially distributed about a mean value. Fourth, we group all alternate/minor/derived alleles while preserving padding columns (Fig. 1f). This further disrupts the AFS, because almost all allele frequencies become zero or one, altering the median allele frequency. Finally, we group all alternate/minor/derived alleles so that reference/major/ancestral alleles blend into any zero-padding (Fig. 1g). This blending removes information about the original number of segregating sites implied by the location of the padding boundary. For methods without zero-padding, we expect these last two permutations (Fig. 1f&g) to be functionally equivalent.

### Application to Published CNNs

For each of the three published studies, we used their respective GitHub repositories (Table S1) to replicate their training and testing procedures. For Torada et al. (2019) and Flagel et al. (2019), we used the provided code to generate training data and to train the models. For Riley et al. (2024), we used a trained model provided by the authors. For all three models, we then used their code and published procedures to generate simulated original test data and to assess model performance on those data. We then applied the permutations described above to the simulated test data and assessed model performance on each level of permuted data. Further details regarding the specific procedure used for each model are in Supplementary Methods.

## Results

### Evaluating CNNs Trained to Detect Positive Selection

Detecting signals of positive selection is one of the most popular population genetics inference tasks. We investigated two CNNs designed for this task: Torada et al. (2019)’s ImaGene and Riley et al. (2024)’s disc-pg-gan. ImaGene is a classic CNN directly trained for the task, while disc-pg-gan is a CNN that was first trained as the discriminator of a generative adversarial network (GAN) developed for demographic history inference (Wang et al. 2021). This discriminator CNN was later fine-tuned with additional training data simulated with selection (Riley et al. 2024). disc-pg-gan performs binary classification (neutral or selection) and ImaGene performs multiclass classification (neutral, moderate selection, strong selection) on input haplotype matrix data.

**Fig. 2.**
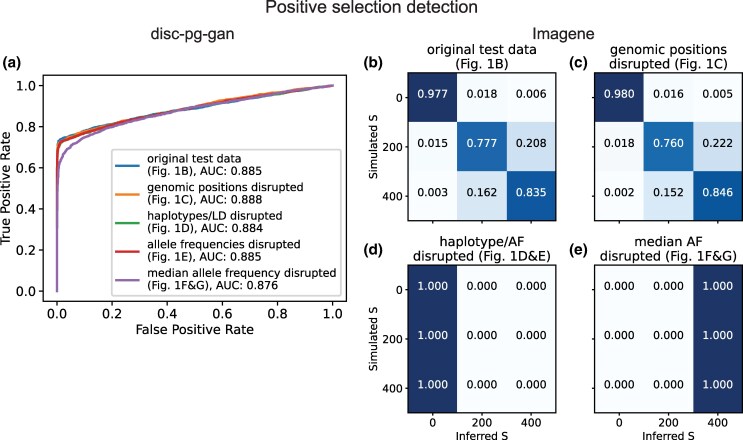
**Performance of positive selection inference CNNs on original and sequentially permuted test data.** a) As measured by area under the receiver operating characteristic (ROC) curve (AUC), the performance of Riley et al. (2024)’s disc-pg-gan network in classifying genomic regions as neutral or positively selected was insensitive to almost all matrix permutations. b)–e) As measured by confusion matrices, the performance of Torada et al. (2019)’s ImaGene network in classifying whether a simulated genomic region was neutral (S=0), under weak-to-moderate positive selection (S=200), or under strong positive selection (S=400), first degraded when haplotypes were disrupted.

The performance of the fine-tuned disc-pg-gan discriminator CNN did not change until the most drastic of our perturbations were applied (Fig. 2a). Because the test data for this network do not require padding, the two most drastic disruptions (Fig. 1f&g) are equivalent, making almost all allele frequencies in the data zero or one. This severe disruption yielded only a small decrease in the area under the receiver operating characteristic (ROC) curve (AUC) (Fig. 2a).

By contrast, performance of the multiclass classifier ImaGene was unchanged when genomic positions were disrupted (Figs. 2b&c, 1c), but when haplotype/LD signals were disrupted (Fig. 1d) the model classified all test data as neutral (Fig. 2d). All test data were also classified as neutral when the AFS was disrupted by permuting all matrix entries (Fig. 1e). At the most drastic disruption levels (Fig. 1f&g), the model classified all input data as experiencing strong positive selection (Fig. 2e).

### Evaluating a CNN Trained to Infer Demographic History

**Fig. 3.**
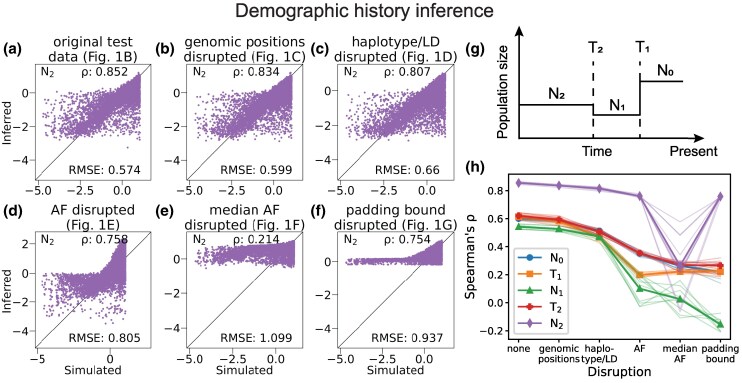
**Performance of Flagel et al. (2019)’s demographic history inference CNN on original and sequentially permuted test data.** The inferred three-epoch demographic history model has five parameters: population sizes $N_{0}$, $N_{1}$, $N_{2}$, and times of an instantaneous size change $T_{1}$ and $T_{2}$. a–f) Scatter plots showing the relationship between the simulated and inferred values when using this CNN to infer the population size parameter $N_{2}$. Each dot represents a simulated test data set, and Spearman’s *ρ* and RMSE scores summarize performance. Scatter plots for the other four parameters are in Fig. S1. g) Illustration of demographic history model inferred. h) Patterns of performance degradation for ten independently trained and tested instances of the CNN. Thin, semi-transparent lines represent individual instances and thicker lines with markers represent the mean across ten instances. Scatter plots shown in panels a–f and Fig. S1 are from one of the ten instances.

To further test the applicability of our permutation method, we applied it to a CNN trained to infer demographic history. The CNN developed by Flagel et al. (2019) is a regressor trained to infer the values of five parameters of a three-epoch demographic history model (Fig. 3g). Among the various CNNs that Flagel et al. (2019) built and tested for this task, this CNN was found to have the best accuracy performance (details in Supplementary Methods).

We applied our permutation test to ten independently trained instances of this CNN architecture. For each instance, new test data were simulated and permuted. Neither the disruption of genomic positions nor of haplotype/LD features had a large effect on performance (Figs. 3a–c, S1a-c). By contrast, when allele frequency signals were disrupted, the CNN performed substantially worse (Figs 3d–f, S1d–f). This trend was consistent for all five parameters and across the ten instances (Fig. 3h).

Among the allele frequency disruptions, generally the more drastic the disruption the worse the performance. Because this CNN includes zero-padding as a preprocessing step, the groupings in Figs. 1f and 1g are distinct. Disrupting the padding led to worse performance for the demographic model parameters $N_{0}$ and $N_{1}$ (Figs. 3e–h, S1e&f). While the accuracy scores (Spearman’s *ρ* and RMSE) for the other parameters do not always follow this trend (Fig. 3h), the scatter plots of predicted versus true values (Figs. 3e&f and S1e&f) show qualitative differences between these two disruption scenarios.

## Discussion

Our permutation method is straightforward to apply, because it does not require model retraining, and it revealed insights about the performance of each of the three models we tested. In general, the results of our permutation method will depend on the simulation regime of the test data. For all three models we examined, and in current typical practice, the test data are generated by a simulation regime that is identical or highly similar to that used to generate the training data. Thus, we draw conclusions about the models themselves from their performance on these permuted test data.

The disc-pg-gan fine-tuned discriminator achieved high AUC score performance without relying on haplotype, LD, or allele frequency features in the test data (Fig. 2a). Because the test data were similar to the training data, the overall high test AUC scores across permutation scenarios suggest that the simulation regime used to generate training data was likely not challenging enough to incentivize the model to learn more complex features. In the test data, the total number of minor allele entries is often drastically lower in the positively selected data matrices compared to neutral matrices (Fig. S2), making it likely the dominant signal. This finding is consistent with Riley et al. (2024)’s result that the pairwise heterozygosity statistic *π* had the highest correlation with the discriminator hidden units out of all the tested summary statistics (their Fig. 3b). This result highlights how our permutation approach can guide method development, by detecting cases in which the simulation regime does not reflect the challenges of real data.

The performance breakdowns of ImaGene when haplotype/LD features are disrupted (Fig. 2d) and of the demographic inference CNN when allele frequencies are disrupted (Fig. 1d) are consistent with population genetic theory expectations. Haplotype structure is commonly used to detect positive selection, and a previous investigation (Cecil and Sugden 2023) of the ImaGene network showed that similar performance can be obtained using just Garud’s H1 statistic (Garud et al. 2015), which is based on LD. Further, grouping all minor alleles (Fig. 1f&g) dramatically reduces observed haplotype diversity, which explains why the inferences shift from all neutrality (Fig. 2d) to all strong selection (Fig. 2e). For the Flagel et al. (2019) demographic inference CNN, haplotype/LD features do not play a substantial role in performance (Fig. 3c&h). This may be due to the small convolutional kernel size of the CNN, spanning only two sites. Such a small kernel size is unlikely to capture long-range patterns of LD. Groupings alleles (Fig. 1f&g) also revealed that the zero-padding itself is among the features learned by the CNN (Fig. 3). This effect of zero-padding has been previously reported for a different CNN developed and studied by Cecil and Sugden (2023) and should be carefully considered when developing CNN training procedures.

Understanding how data transformations affect model performance is broadly important in machine learning. Often, it is desirable that a model be invariant to certain transformations. For example, in population genetics the order of haplotype rows in a data matrix is not biologically meaningful, so models can be explicitly designed to be invariant to row permutations (Chan et al. 2018; Sanchez et al. 2021; Wang et al. 2021). More broadly, theoretical work in deep learning and computer vision has characterized model architectures that are invariant to certain transformations (Korshunova et al. 2018; Bloem-Reddy and Teh 2020). For models that are not explicitly invariant, data augmentation with transformed data, such as skewed or color-shifted images, can result in effective invariance and improved performance (Shorten and Khoshgoftaar 2019; Chen et al. 2020), especially when training data are limited. Our post hoc testing approach is not focused on enforcing expected invariance but rather on interpreting model performance. We thus do not test transformations that population genetics models are expected to be invariant to (such as permuting haplotype matrix rows). Rather, we test permutations to which models cannot be assumed *a priori* to be invariant. We interpret sensitivity to a given permutation to mean that a corresponding population genetic feature is important for inference.

Our haplotype matrix permutation approach contributes to the growing body of work that aims to derive deeper insights from population genetics machine learning models beyond performance metrics, as these models can be tools for learning how to solve a task rather than just for completing the task (Cecil and Sugden 2023). Understanding which features primarily drive performance can inform new theory (e.g. Hahn and Mishra 2025’s finding that the AFS can be used to estimate recombination) and inform the development of more efficient methods by focusing only on the most salient features (e.g. Cecil and Sugden 2023’s finding that ImaGene’s performance can be achieved using just Garud’s H1 statistics Garud et al. 2015). Our permutation approach provides a simple framework to derive similar insights, and it can be applied to any methods that use haplotype matrices as input, such as the image processing approach developed by Amin et al. (2024).

## Supplementary Material

msaf250_Supplementary_Data

## Data Availability

All code used for analysis in this paper is available at https://github.com/lntran26/ConfuseNN.
