# Supplementary material for: Interpreting Supervised Machine Learning Inferences in Population Genomics Using Haplotype Matrix Permutations
Source: Mol Biol Evol. 2025 Oct 6;42(10):msaf250. doi: 10.1093/molbev/msaf250 (PMC12548731; doi:10.1093/molbev/msaf250)
Supplement: msaf250_Supplementary_Data [file msaf250_supplementary_data.pdf]

Supplementary Methods

Table S1: Github repositories of published CNNs evaluated

Study	Github repository
Riley et al. (2024)	`https://github.com/mathiesonlab/disc-pg-gan`
Torada et al. (2019)	`https://github.com/mfumagalli/ImaGene`
Flagel et al. (2019)	`https://github.com/flag0010/pop_gen_cnn/tree/master/demography`

Positive selection classifier CNN - disc-pg-gan

Riley et al. (2024) trained several discriminators using different human populations and random seeds. We evaluated discriminator CNN seed 19 trained on CEU population parameters provided by the authors and available at `https://github.com/lntran26/ConfuseNN`. This CNN has been fine-tuned with positive selection simulations generated using SLiM (Haller & Messer 2019).

To generate simulated test data, we used the SLiM-based procedure Riley et al. (2024) employed to simulate fine-tuning data. Similar to the original work, we also generated 3,000 simulated genomic regions for the neutral case (true negative in the binary test) and 2,400 simulated genomic regions for positive selection case (true positive), using four positive selection strengths (selection coefficient $s = 0.01, 0.025, 0.05, 0.1$), each contributing 600 simulated genomic regions.

Positive selection classifier CNN - ImaGene

Training and test simulations were generated with *msms* (Ewing & Hermisson 2010) using a three-epoch demographic history model with specified parameters from (Marth et al. 2004) for the CEU population, following Torada et al. (2019). The simulated genomic regions were of size 80 kb, and 128 haplotypes were sampled from CEU individuals. The demographic history model has two instantaneous population size change events: a bottleneck from $10,000$ to $2,000$ individuals at 3500 generations before the present, and a recovery and expansion to $20,000$ individuals at 3000 generations before the present. 5000 simulations were generated per selection coefficients $S = 0, 200, 400$ in $2N$ units, corresponding to $S = 0, 0.01, 0.02$.

Each simulation was processed into a 128×128 haplotype matrix using major/minor allele

polarization and removal of loci with less than 1% allele frequency. The 128 rows in the final matrix correspond to the 128 sampled haplotypes, while the variable number of segregating sites were standardized into 128 with the image resizing Python package *scikit-image* (Van der Walt et al. 2014). Both rows and columns of the haplotype matrices were sorted by frequency of occurrence.

The network used here was implemented using Python packages TensorFlow v2.15.0 and Keras v2.15.0. The network architecture consists of three convolutional layers. Each layer has 32, 64, and 64 filters, respectively, with a ReLU activation function followed by a max pooling layer. The output is flattened before being passed into the final output dense layer with 3 nodes, corresponding to the 3 classification classes ($S = 0, 200, 400$) and a softmax activation function.

Demographic history inference CNN

Training and test simulations were generated with *ms* (Hudson 2002) using a three-epoch demographic history model as described in Flagel et al. (2019). Briefly, 100,000 1.5MB regions were simulated with human mutation and recombination rates. The demographic history model parameters were sampled from ranges described in Table S2. As done in the original study, we also divided the mutation rate by 10, which is equivalent to randomly downsampling the total number of SNPs by a factor of 10. 80,000 simulations were used in training and 20,000 simulations were used for accuracy testing as well as the permutation test.

Each simulation was processed into a haplotype matrix with 50 rows, corresponding to the number of sampled haplotypes. The width of the matrix equals the maximum number of segregating sites observed across all simulations. For simulations with fewer sites, columns of zeros were added to the right edges (zero-padding) to make all matrices have the same fixed width.

The position vector has length equal to the maximum number of segregating sites as above minus one. Each value in this vector is the normalized distance between two adjacent segregating sites, with 1 being the total length of the 1.5 MB simulated region. Position vectors were also zero-padded so they all have the same length.

The network used here was implemented using Python packages TensorFlow v1.1.0 and Keras v2.0.6. This network has two branches, one takes the haplotype matrix as input and the other takes

the position vector as input. The haplotype matrix branch consists of four convolutional layers, each with 128 filters followed by a max pooling layer. The position vector branch consists of a dense layer with 32 nodes. The output of these two branches are concatenated before being fed into the final dense layer of 256 nodes. We implemented the best performing hyperparameters reported in the original study: 1D convolution type, kernel size 2, output in log scale, drop-out layers throughout, and with sorting of chromosomes by genetic similarity.

Supplementary Results

Table S2: Our accuracy scores compare to the original work in Flagel et al. (2019) when replicating the demographic history inference CNN.

	Flagel et al.	Our replication	Flagel et al.	Our replication
Parameter (range)	RMSE	RMSE	Spearman's ρ	Spearman's ρ
$N_0(100, 40000)$	N.A.	0.584	0.59	0.591
$N_1(100, 5000)$	N.A.	0.764	0.54	0.544
$N_2(100, 20000)$	N.A.	0.571	0.84	0.856
$T_1(100, 3499.99)$	N.A.	0.741	0.62	0.605
$T_2(T_1 + 1, T_1 + 3500)$	N.A.	0.674	0.62	0.609
RMSE on validation set	0.43366	0.451		

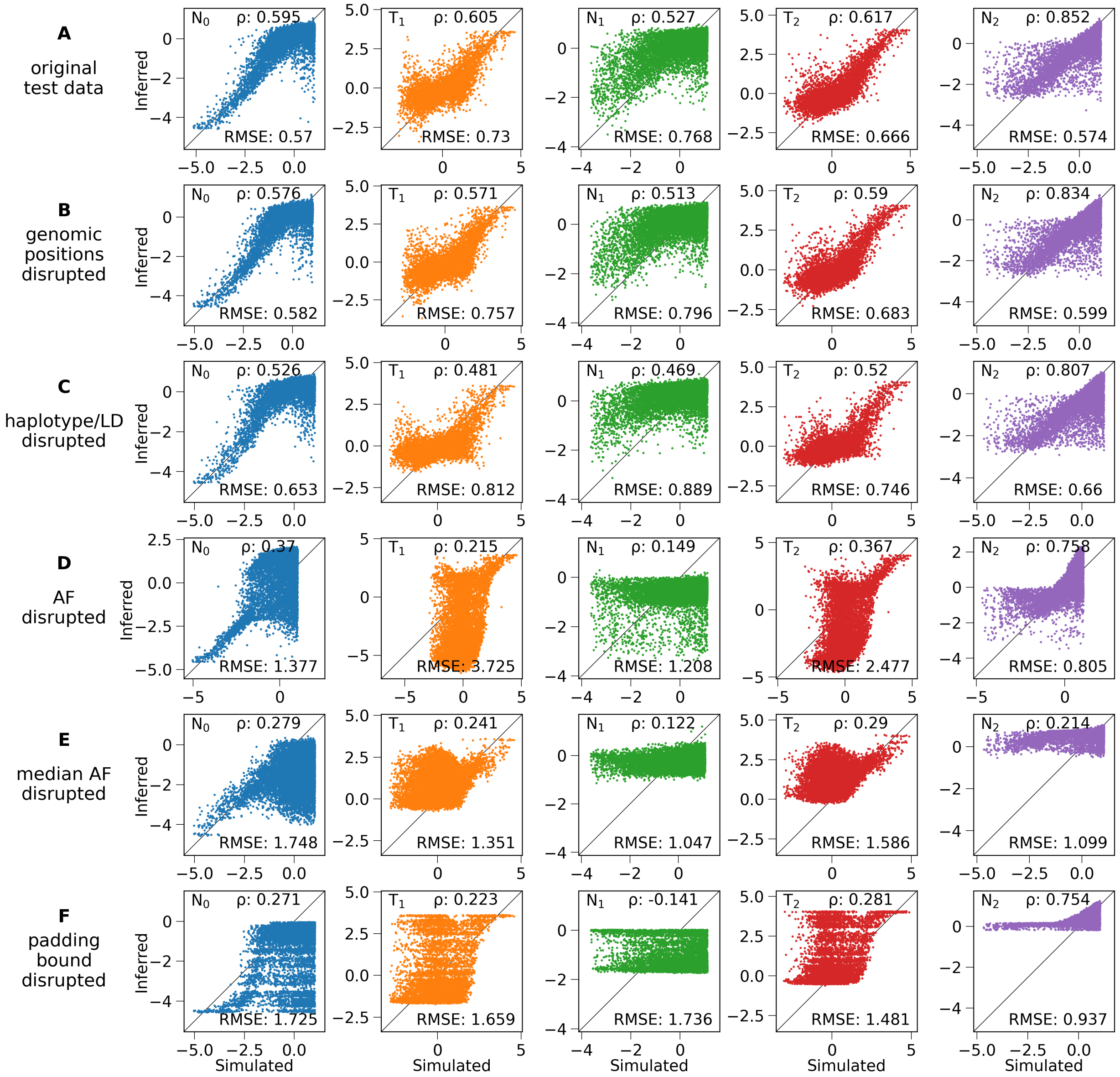

Figure S1: **Performance of Flagel et al. (2019)'s demographic history inference CNN on original and sequentially permuted test data for all five demographic history model parameters.** (A) Replicating the original CNN's performance using the original test data. (B-F) The accuracy of the original CNN when tested on different permuted test data shown in Fig. 1. Accuracy is measured by the Spearman's rank coefficient ρ and the root mean squared error (RMSE) as per the original work. All parameter values are in log scale.

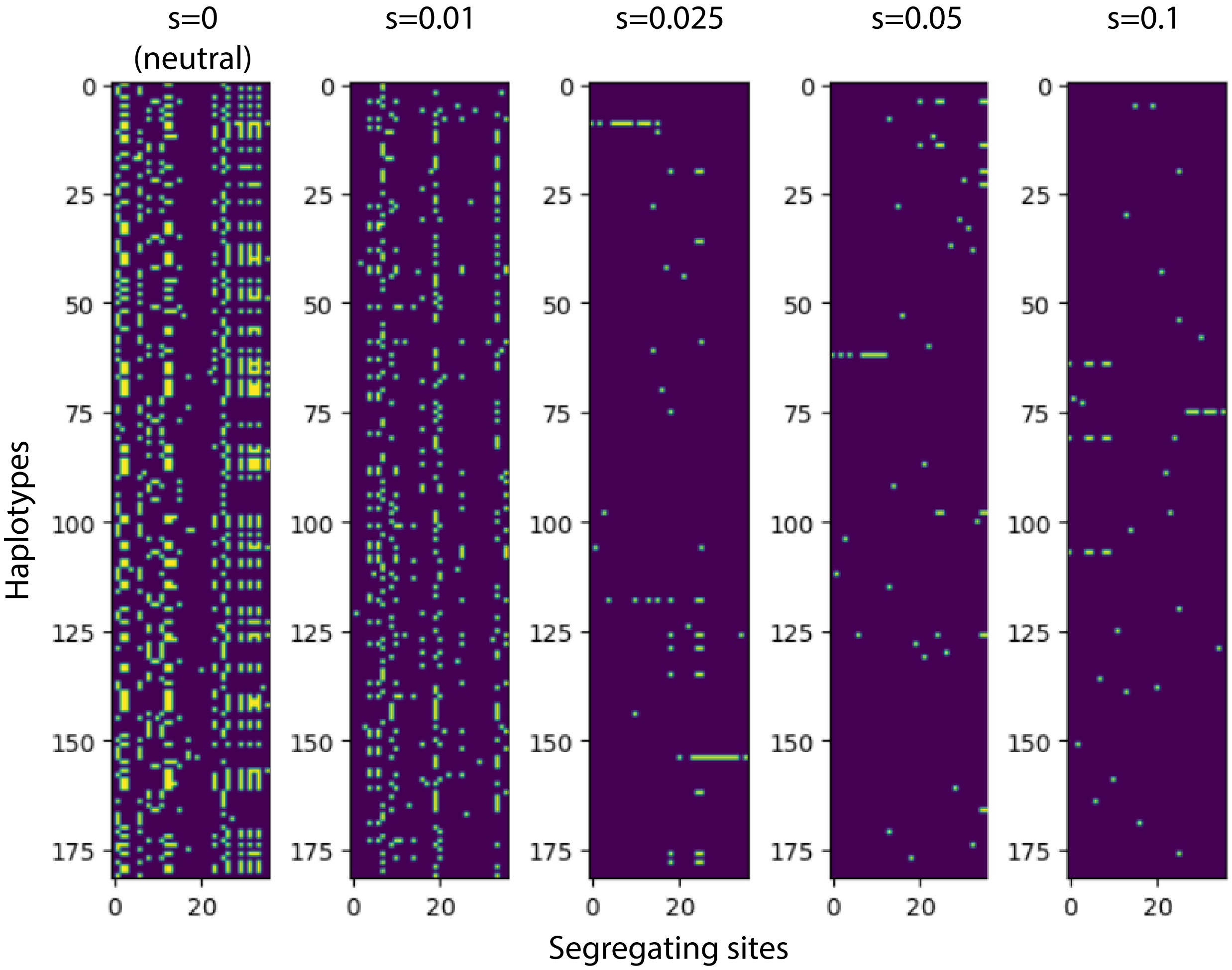

Figure S2: **Example of the qualitative difference in test data sets used for fine-tuning disc-pg-gan.** The total number of minor allele entries is often drastically lower in the positively selected haplotype matrices ($s = 0.01, 0.025, 0.05, 0.1$) compared to the neutral matrix ($s = 0$).

REFERENCES

Ewing G, Hermisson J (2010) MSMS: a coalescent simulation program including recombination, demographic structure and selection at a single locus. Bioinformatics 26:2064.

Flagel L, Brandvain Y, Schrider DR (2019) The unreasonable effectiveness of convolutional neural networks in population genetic inference. Molecular Biology and Evolution 36:220.

Haller BC, Messer PW (2019) SLiM 3: forward genetic simulations beyond the Wright–Fisher model. Molecular Biology and Evolution 36:632.

Hudson RR (2002) Generating samples under a Wright–Fisher neutral model of genetic variation. Bioinformatics 18:337.

Marth GT, Czabarka E, Murvai J, Sherry ST (2004) The allele frequency spectrum in genome-wide human variation data reveals signals of differential demographic history in three large world populations. Genetics 166:351.

Riley R, Mathieson I, Mathieson S (2024) Interpreting generative adversarial networks to infer natural selection from genetic data. Genetics 226:iyae024.

Torada L, Lorenzon L, Beddis A, Isildak U, Pattini L, Mathieson S, Fumagalli M (2019) Imagene: a convolutional neural network to quantify natural selection from genomic data. BMC Bioinformatics 20:337.

Van der Walt S, Schönberger JL, Nunez-Iglesias J, Boulogne F, Warner JD, Yager N, Gouillart E, Yu T (2014) scikit-image: image processing in Python. PeerJ 2:e453.
